# Nano- and Microcarriers as Drug Delivery Systems for Usnic Acid: Review of Literature

**DOI:** 10.3390/pharmaceutics12020156

**Published:** 2020-02-15

**Authors:** Ana Zugic, Vanja Tadic, Snezana Savic

**Affiliations:** 1 Institute for Medicinal Plant Research “Dr. Josif Pancic”, Tadeusa Koscuska 1, 11000 Belgrade, Serbia; azugic@mocbilja.rs; 2 Department of Pharmaceutical Technology and Cosmetology, Faculty of Pharmacy, University of Belgrade, 11000 Belgrade, Serbia; snexs@pharmacy.bg.ac.rs

**Keywords:** usnic acid, nanocarriers, microcarriers, delivery systems, natural products

## Abstract

Usnic acid is one of the most investigated lichen secondary metabolites, with several proven biological properties with potential medical relevance. However, its unfavorable physico-chemical properties, as well as observed hepatotoxicity, have discouraged wide-range utilization of usnic acid as a promising therapeutic agent. In accordance with the growing research interest in the development of nanotechnology, especially in the arena of preparations based on natural sources of medicinal compounds, usnic acid incorporated into nano- and microsized colloidal carriers has been a subject of a large number of publications. Therefore, this review discusses the overall results of the studies dealing with usnic acid encapsulated into lipid-based, polymeric and nonorganic micro- and/or nanocarriers, as potential drug delivery systems for this natural compound, in an attempt to introduce its usage as a potential antitumor, antimicrobial, wound-healing, antioxidative and anti-inflammatory drug.

## 1. Introduction

Investigations of biological activities of lichens and their secondary metabolites with potential medical relevance over the past decades have singled out usnic acid as one of the most prominent lichen substances. Usnic acid was the subject of a vast majority of studies, including the ones discussing this substance as an isolated compound [1] or as a part of the usnic acid-containing lichens extracts [2,3,4,5], as well as investigations dealing with chemical derivatives of usnic acid [6]. As the stated studies suggested, usnic acid or its derivatives possess various biological activities, such as antimicrobial against various bacteria, fungi and parasites, as well as antioxidant, cytotoxic (antitumor), neuroprotective, gastroprotective, cardioprotective, cytoprotective, immunostimulatory, anti-inflammatory and wound healing properties [1]. Nevertheless, hepatotoxicity of this substance after systemic administration was later revealed. Its poor water solubility discouraged wide-range application of usnic acid as a promising therapeutic agent, mirrored in the lack of commercially available products containing usnic acid as an active substance, with the exception of the scarce number of products for topical application containing this compound as an active ingredient, among several other activities (Table 1).

**Figure 1:**
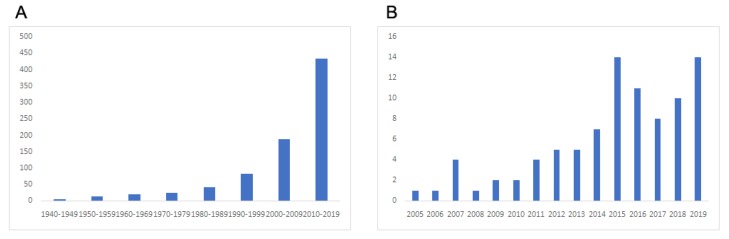
Pattern of scientific research on usnic acid found on Scopus: (**A**) from 1940–2019 (search was restricted to the term “usnic acid” in the abstract), (**B**) from 2005–2019 (search was restricted to the term “usnic acid” and “nanoparticles” and “microparticles” in all fields).

A renewed interest in usnic acid in recent years has been reflected not only in the growth of the number of publications (Figure 1), but also in several review articles dealing with its chemical [6] and biological properties, its toxicity [1,7] and also its potential pharmaceutical use [8]. With respect to the later, the present review focuses on the development of drug delivery systems (DDSs) aiming to stress the ways to overcome stated drawbacks, thereby increasing the therapeutic index of this drug by improving the efficacy and/or reducing the toxicity. Considering the growing research interest in the development of nano- and microsized colloidal carriers, especially in the arena of preparations based on natural sources of medicinal compounds [9,10], special interest is given to nano- and microparticulate formulations containing usnic acid.

Being aware of the shortage of the comprehensive reviews regarding nano- and microsized carriers of usnic acid as active substance, this paper was designed to describe the overall results of the studies dealing with usnic acid encapsulated into lipid-based nanocarriers, polymeric nanocarriers/microparticles and nonorganic nanoparticles, as DDSs for this natural compound, in an attempt to introduce its usage as a potential antitumor, antimicrobial, wound-healing, antioxidative and anti-inflammatory therapeutic agent.

## 2. Lipid-Based Nanocarriers

### 2.1. Liposomes

**Figure 2:**
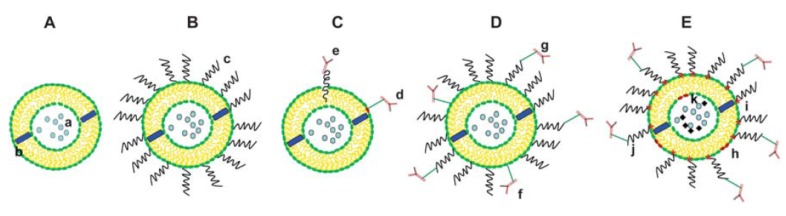
Evolution of liposomes. (**A**) Early conventional ‘plain’ phospholipid liposomes with water-soluble drug (a) entrapped within the aqueous liposome interior, and water-insoluble drug (b) incorporated in the liposomal membrane. (**B**) Long-circulating liposome grafted with a protective polymer (c) such as polyethylene glycol (PEG), which shields the liposome surface from the interaction with opsonizing proteins. (**C**) Antibody-targeted immunoliposome with antibody covalently coupled (d) to the reactive phospholipids in the membrane, or hydrophobically anchored (e) in the liposomal membrane after preliminary modification with a hydrophobic moiety. (**D**) Long-circulating immunoliposome simultaneously bearing both a protective polymer and an antibody (f) or, preferably, antibody bound to the distal end of a grafted polymeric chain (g). (**E**) “Smart” liposome, the surface of which can be modified (separately or simultaneously) by the incorporation of stimuli-sensitive lipids such as temperature, pH, reduction sensitive lipids (h); modification with a detachable stimuli-sensitive protective polymer (i) or modification with a detachable stimuli-sensitive protective polymer with a targeting ligand such as an antibody attached to the distal tip of the polymer (j); loading with magnetic particles (k) for magnetic targeting. Republished with permission of [Royal Society of Chemistry], from [Liposomes as ‘smart’ pharmaceutical nanocarriers, Sawant, R.R. and Torchilin, V.P., 6, 17, 2010] [14]; permission conveyed through Copyright Clearance Center, Inc.

In general, liposomes have been the most successful DDSs among all the investigated nanocarriers [13]. A schematic presentation of evolution of liposomes is presented in Figure 2. Out of all modifications of liposomes, most of them were dedicated to passive targeting enabling increased drug accumulation in the target tissue via the so-called enhanced penetration and retention (EPR) effect. In addition, liposomes have been investigated for active targeting, stimuli-responsive controlled release, and also enhanced cellular penetration and the increase of solubility of highly insoluble drugs [14]. 

Usnic acid has been incorporated into liposomes, mostly for the improvement of antimicrobial activity (alone or in combination with antibiotics), using either conventional or surface-functionalized liposomes. The later have also been investigated for the enhancement of antioxidant activity. Also, liposomes loaded with usnic acid incorporated into polymeric-based thin films were investigated as potential wound dressings (Table 2). Details of studies concerning usnic acid loaded into nanoliposomes are presented below. 

In the pioneer paper on liposomes containing usnic acid reported by Lira et al., conventional liposomes were chosen as suitable carriers of usnic acid for its targeted delivery into macrophages, as reservoirs of *Mycobacterium tuberculosis* (Mbt), in a contribution to a potential usage of this drug in the treatment of human pulmonary tuberculosis [15]. Among several investigated formulations, the one of positively charged usnic acid-loaded liposomes composed of 42 μmol of lipids/10 μL of buffer and 1.5 mg/mL of usnic acid (drug: lipid ratio = 1:12) was chosen for further investigations as the formulation with the highest drug load and good preliminary stability (Table 2). Further lyophilization of this formulation enabled its excellent long-term stability after 24 months of storage. The high encapsulation efficiency and the great stability of the prepared liposomes (Table 2) was explained by unipolar interactions associated with the hydrophobic forces of this molecule and phosphatidylcholine bilayers, but also the presence of a positively charged lipid in the bilayer (stearylamine). Such a finding was substituted by the fact that neutral and negatively charged liposomes loaded with usnic acid, which were investigated in the preformulation study, were found to be unstable immediately after preparation. The same interactions were speculated to control the release of usnic acid from the liposomes, which was assessed in vitro using dialysis techniques (acceptor medium: phosphate buffer, pH 7.4). Namely, the in vitro liberation profile was shown to be bimodal, where an initial burst effect (20.6 ± 0.4% of released drug in the first 8h) was connected to the fraction of usnic acid located at the outer monolayer of the phospholipid bilayer, while further slow release of usnic acid, described by the first-order kinetics (from the 8^th^ to 72^nd^ hour), was attributed to the drug fraction located at the inner phospholipid monolayer of the liposomal bilayer. An improvement in the intracellular uptake of the liposomes by the J774 murine macrophages (21.6 × 104 ± 28.3 × 102:9.5 × 104 ± 11.4 × 102 c.p.s. for free usnic acid) and its longer maintenance inside macrophages were observed using fluorescence spectroscopy, pointing to facilitation of usnic acid penetration into the cells. The in vitro investigation of antimicrobial activity of free and liposome-loaded usnic acid was assessed by means of the microplate alamar blue assay, used for the determination of the minimal inhibitory concentration (MIC) and the minimal bactericidal concentration (MBC). The obtained MIC and MBC values for free usnic acid were higher than that for the usnic acid encapsulated into the liposomes (6.5 and 5.8 μg/mL, and MBC of 32 and 16 μg/mL, respectively). However, they were not as different as expected, based on previously observed increased uptake of the liposomes within macrophages, compared to the free drug. Such results were explained by the well-known defense mechanism of pathogenic mycobacteria that inhibit fusion between phagosomes and lysosomes that might have decreased the release of the drug from the liposomes. Cytotoxicity of the prepared liposomes was assessed using an MTT assay in the J774 cell line and was expressed as the percentage of cell viability, calculated with respect to the untreated control cells. Unloaded liposomes, used as a negative control, showed no cytotoxic effect. On the other hand, liposomes loaded with usnic acid revealed stronger cytotoxic activity compared to pure usnic acid (IC_50_ values being 12.5 ± 0.26 μg/mL and 22.5 ± 0.60 μg/mL, respectively). These findings were related to the enhanced macrophage uptake of usnic acid loaded into liposomes compared to the free drug. 

In a further attempt to increase water solubility of usnic acid, Lira et al. [17] prepared inclusion complexes with β-cyclodextrin (β-CD) by means of freeze-drying prior to encapsulation of either the prepared complex or free usnic acid into liposomes intended for targeted antimicrobial activity of this drug. Interactions between usnic acid and β-CD, detected by spectroscopic analysis (IR and NMR spectroscopy, X-ray powder diffraction and thermal analysis), indicated the formation of an inclusion complex (designated as UA: β-CD) that led to an increase in water solubility of usnic acid (5-fold increase in solubility at 37 °C in the presence of 16 mM (1.8%) of β-CD), but it did not affect its antimicrobial activity, since inhibitory zone diameters assessed using the agar disc diffusion method for the UA: β-CD and free usnic acid were the same for all the tested microorganisms (*Streptococcus mutans*, *Enterococcus faecalis* and *Aggregatibacter actinomycetemcomitans*). Incorporation of UA: β-CD into liposomes (marked as UA: β-CD-Lipo) prepared in the same manner as in their previous study [15] was limited to drug loading of 1.2 mg/mL (12:1 lipid:drug molar ratio, i.e., 42 mM of lipids) corresponding to 10 mM of usnic acid in the cyclodextrin complex (UA: β-CD) due to the noted instability of liposomes in higher complex concentrations ascribed to the destabilizing action of cyclodextrin on the liposomal membrane. The obtained liposome formulation loaded with usnic acid-cyclodextrin complex (UA: β-CD Lipo) was stable for 5 months after lyophilization and released usnic acid more slowly compared to the usnic acid-loaded liposomes (UA-Lipo). This was further connected to the lower antimicrobial activity of UA: β-CD Lipo against methicillin-resistant *Staphylococcus aureus* (MRSA), assessed using the agar diffusion well method. Namely, usnic acid (tested in the concentrations of 300, 600 and 1200 µg/mL) was effective against two out of three investigated MRSA strains at 600 µg/mL compared to tetracycline (300 µg/mL), used as positive control. Usnic acid encapsulation into liposomes did not improve its antimicrobial activity, since UA-Lipo was effective against the same MRSA strains only at higher concentrations (1200 µg/mL), whereas UA:β-CD-Lipo was ineffective against all tested MRSA strains even at the highest investigated concentration. Better antimicrobial activity of liposomes loaded with usnic acid (UA-Lipo) compared to the ones loaded with UA:β-CD (UA:β-CD-Lipo) was explained by the short time of incubation in the experimental setup of antimicrobial susceptibility assessment (which was 72 h, allowing release of only around 30% of usnic acid from UA: β-CD Lipo, compared to around 70% from UA-Lipo).

In a recent study of the same research group [16], the antimicrobial effects of usnic acid encapsulated into liposomes against resistant strains of Mbt were investigated in combination with rifampicin and isoniazid as the first-line drugs in the treatment of tuberculosis. For this purpose, an in vitro microplate alamar blue assay test was employed for the evaluation of MIC values against several clinical isolates of Mbt, for which resistance to rifampicin and isoniazid was previously confirmed. Moreover, in vitro interactions between rifampicin/isoniazid and usnic acid (free and encapsulated into liposomes) were evaluated by the checkerboard bidimensional method. In comparison to their first study [15], Ferraz-Carvalho et al. prepared positively charged liposomes using the same lipids (Table 2) but in different amounts, leading to higher lipid:drug molar ratio (14:1) and increased drug loading (2 mg/mL). However, the effect of higher concentrations of usnic acid loaded into the prepared liposomes on their stability was not investigated. Encapsulation of usnic acid into liposomes led to 30-fold lower MIC values for all tested clinical isolates compared to free usnic acid (0.98:31.25, respectively). Such results were explained by possible electrostatic interactions between negatively charged carboxyl groups of mycolic acids in the Mtb cell wall and positively charged liposomal vesicles, or the direct interaction of liposomes and bacteria by the fusion processes, leading to the release of the encapsulated drug within the bacteria [16]. Also, both free and encapsulated usnic acid exhibited a synergistic effect with rifampicin in vitro using the checkerboard method (fractional inhibitory concentration index, FICI < 0.5), unlike the combinations with isoniazid that showed an indifferent effect (1 < FICI ≤ 4). 

In a similar scenario investigating combined therapy against resistant bacteria, two new compounds of natural origin, usnic acid and β-lapachone (free and liposome-loaded), were tested alongside vancomycin for their antimicrobial effects against 10 clinical isolates of MRSA, using in vitro microdilution for the determination of MIC and MBC values [18]. Liposomes loaded with usnic acid were prepared according to Lira et al. [15]; therefore, a high encapsulation efficiency of this compound was achieved, together with low particle size (Table 2), an acceptably low PDI (0.362) and positive zeta potential (ξ = +11.8 ± 0.5). Encapsulation of usnic acid into nanoliposomes potentiated its bacteriostatic effects towards tested MRSA strains, considering the decrease of MIC from 8–32 to 4–8 μg/mL for the free and liposome-loaded drug, respectively. The same could not be stated for MBC values, which were equivalent or even lower for the free usnic acid compared to usnic acid incorporated into liposomes, with the exception of one tested MRSA strain. In addition, the interaction study evaluated by the checkerboard method suggested usnic acid to be a possible promoter of the antimicrobial activity of vancomycin. This was especially pronounced in the case of usnic acid loaded into liposomes, which revealed synergism with vancomycin (FICI < 0.5), compared to the free drug showing an additive interaction with vancomycin (0.5 ≤ FICI ≤ 1).

**Figure 3:**
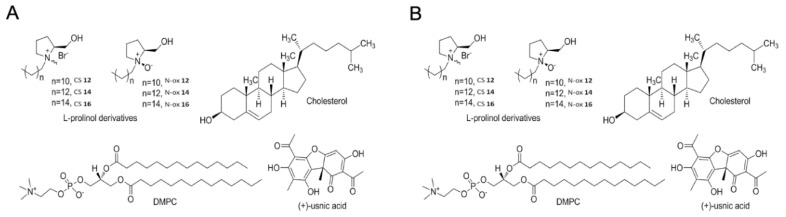
Molecular structure of liposome components and of usnic acid (UA) used in (**A**) study of Francolini et al. (Reprinted from Colloids and Surfaces B: Biointerfaces, 181, Francolini, I., Giansanti, L.; Piozzi, A.; Altieri, B.; Mauceri, A.; Mancini, G, Glucosylated liposomes as drug delivery systems of usnic acid to address bacterial infections, 632–638 [19], Copyright (2019), with permission from Elsevier), and (**B**) a study of Battista et al. (Reprinted from Colloids and Surfaces A: Physicochemical and Engineering Aspects, 585, Battista, S.; Campitelli, P.; Galantini, L.; Köber, M; Vargas-Nadal, G.; Ventosa, N.; Giansanti, L, Use of N-oxide and cationic surfactants to enhance antioxidant properties of (+)-usnic acid loaded liposomes, 124154 [20], Copyright (2020), with permission from Elsevier).

Two recently published papers discussed the usage of positively charged liposomes, which were functionalized in order to enhance biological effects of the encapsulated usnic acid [19,20]. To this end, physicochemical characterization of the produced liposomes was performed, and afterwards the influence of the lipid components used for their production (differing in chain length, size and charge of their headgroups, saturation or substituents) and/or method of usnic acid loading to the biological activities of usnic acid was investigated. In particular, Francolini et al. [19] investigated the possibility of target delivery of usnic acid-loaded cationic nanoliposomes to biofilms of *Staphylococcus epidermidis*. For this reason, glycosidic moieties were introduced to the liposome surface, aiming to exploit the presence of glucose transporters on *S. epidermidis* and, therefore, increase liposomes selectivity and subsequent accumulation of usnic acid in this bacterium. Therefore, investigated liposomes were prepared using 1,2-dioleoyl-sn-glycero-3-phospholcholine (DOPC), cholesterol (chol) and one of the three structurally related glucosylated amphiphiles (GA1, GA2 and GA3; Figure 3a). Thereby, GA1 and GA2 differ in the length of the hydrophilic spacer linking the glucose residue to the quaternary ammonium group (Figure 3a). Also, aside from being carriers of the glycosidic moiety, both GA1 and GA2 were chosen for this investigation as positively charged molecules, as it was previously shown that biofilms of *S. epidermidis* were negatively charged. Liposomes prepared with GA1 and GA2 were designated DOPC/chol/GA1 or DOPC/chol/GA2, respectively. In this connection, it was also of interest to elucidate the contribution of the mere sugar residues or positive charge on the surface of the investigated liposomes on their physico-chemical properties and biological effects. Therefore, liposomes were also prepared with GA3 (designated DOPC/chol/GA3) as the neutral pH-sensitive analogue of GA1 (GA 3 being a molecule with sugar moiety, but without positive charge) and cetyltrymethyl ammonium bromide, CTAB (designated DOPC/chol/CTAB), as the mere cationic amphiphile. Also, liposomes prepared only with DOPC and cholesterol (devoid of both positive charge and sugar residues, designated DOPC/chol) were included in this investigation. Usnic acid encapsulation into the liposomes designated as DOPC/chol, DOPC/chol/GA1 and DOPC/chol/GA2 was performed by two passive loading techniques (during liposomes formation, protocol A, or on preformed liposomes, protocol B), as well as using active loading. It was found that the type of the used glucosylated amphiphile did not influence the entrapment efficiency, which could not be stated for the method of usnic acid incorporation. Namely, the highest entrapment efficiency (≈85%) was achieved using protocol B, which was unexpectedly higher compared to the active loading technique (entrapment efficiency ≈65%). Such a finding was explained by the lower water solubility of usnic acid due to the higher percentage of its prisonization in the lower pH used in this active loading protocol. Based on these results, usnic acid loading on preformed liposomes (protocol B) was used for the production of the formulations DOPC/chol/GA3 and DOPC/chol/CTAB. Afterwards, the anti-biofilm activity of all the investigated liposomes was evaluated versus a standard strain of *S. epidermidis* (ATCC 35,984), at MIC concentration of free usnic acid (16 μg/mL) and fivefold less than MIC, the rationale for the latter being expected enhancement of the usnic acid efficacy after incorporation into liposomes. It was shown that liposomes without usnic acid (negative control) did not show any antimicrobial action. Also, encapsulation of usnic acid into liposomal formulations did not improve its antibacterial action, except in the case of DOPC/chol/GA1(2) liposomes, in both tested concentrations. These results were confirmed by SEM, suggesting that both sugar moiety and positive charge on the liposome surface have a synergic effect in promoting its interaction with the investigated microorganism.

In another study, impact of an N-oxide moiety in the phospholipid bilayer to the antioxidative activity of usnic acid entrapped in the liposomes was investigated, bearing in mind that the presence of N-oxide in the molecule structure was found to mimic the activity of superoxide dismutase, and also that aggregates of surfactants carrying this moiety can affect the antioxidant activity of a molecule they include [20]. Additionally, the effect of the presence of the quaternary ammonium moiety, instead of N-oxide, and also different chain lengths (C12, C14 and C16) in both groups of ligands attached to the surface of liposomes loaded with usnic acid were assessed (Figure 3b). The authors found that different chain length and/or charge of the polar headgroup of the used amphiphiles had an impact on the zeta potential, entrapment efficiency and in vitro antioxidant activity, concluding that, more than the charge or the chain length, the combination of both these parameters was fundamental in affecting liposomes physicochemical properties and their ability of influencing the antioxidant effectiveness of usnic acid. 

Although both studies indicated incorporation of usnic acid into functionalized nanoliposomes to enhance its biological properties, the investigated liposomal suspensions were found to be stable for at least two weeks [19] or less [20], and this issue should be addressed prior to their potential practical usage in the treatment of infections/oxidative stress-related diseases. 

The research group of Nunes et al. investigated usnic acid-loaded liposomes into polymeric i.e., collagen [21,22] and gelatin-based films [23], addressing their potential usage as wound dressing for dermal burn healing assessed in an in vivo porcine model. In the later study [23], liposomes were used as carriers of usnic acid, enabling its controlled delivery into the burning site, while gelatin-based matrices were used as dressing membranes for (+)-usnic acid-loaded liposomes. Indeed, an in vitro liberation study of usnic acid from the membranes revealed a controlled release between the 4^th^ and 24th hour of the experiment, reaching 98.15% of the released drug after the initial burst effect in the first 4 h. Such a release profile of usnic acid from the liposome-loaded gelatin-based films correlated well with the content of this substance determined in the epidermis and dermis in an in vitro penetration/permeation study conducted using porcine ear skin as the membrane. Although the earlier results revealed the wound healing ability of usnic acid derivatives in an in vitro wound healing model consisting of monolayers of HaCaT keratinocytes, to examine the wound healing potential of usnic acid-loaded liposomes, Nunes et al. employed in vivo assay in their study, namely the linear incision wound model, with the measurements of the wound healing parameters on 8, 18, and 30 days. After confirming that in a macroscopic assay any clinical signs of secondary infections could not be observed, microscopy after 8 days showed hydropic degeneration of the epithelium, with intense neutrophilic infiltration; at 18 days, the epidermal neo-formation was partial, with exuberant and cellularized granulation tissue. At 30 days, observed restricted granulation tissue in the region below the epithelium was significant, with greater collagen density. The obtained results gave insight into possible development and maturation of granulation tissue and scar repair by investigated usnic acid-loaded liposomes, with the important findings of satisfactory collagen deposition. The suggested mechanism of wound healing action of usnic acid-loaded liposomes might be through its large capacity to promote cell motility. Interestingly, at the end of the experiment, the treated wounds were in an advanced healing phase, confirming the ability of this compound to effect the inflammatory response during wound healing, attenuating the expression of TNF-a, IL-6, IL-8 and macrophage inflammatory protein-2 (MIP-2), as well as improving the level of IL-10. Taking into account the previous studies using rodent models, which provided strong evidence that usnic acid mixed with film collagen matrices had positive modulatory action on the synthesis of scar collagen [22], the use of gelatin membranes containing usnic acid loaded-liposomes in this study pointed out the healing and macroscopic shrinkage of the wound due the controlled release of drug. Further direction might be to investigate the effects that usnic acid-loaded liposomes incorporated into polymers might exhibit in healing the wounds compromised by secondary bacterial infections, taking into account the great antimicrobial potential of this compound.

### 2.2. Nanoemulsions

Nanoemulsions are thermodynamically unstable mixtures of oil and water, with one of the liquids being dispersed in the other (water-in-oil or oil-in-water) forming very small (submicron) droplets, stabilized by a surfactant. Thereby, oil-in-water nanoemulsions are more commonly used as drug carriers, aiming to encapsulate lipophilic drugs in aqueous environment. In recent years, a growing interest in nanoemulsions research could be observed, owing to their potential for their high solubilization capacity for hydrophobic activities, protection of unstable compounds and enhanced delivery and tissue-penetrating properties associated with good stability and relative easiness of preparation [9,10].

This subject has been investigated by Mukerjee et al., who prepared nanoemulsions containing usnic acid dispersed in cinnamon oil (1:4 ratio), which was used as the oil phase [24]. Nanoemulsions were prepared by means of ultrasonic emulsification, and (based on the previous solubility study of cinnamon oil-usnic acid mixture (CUB)) Tween 80 and ethanol were chosen as surfactant and co-surfactant, respectively. Further, the ratio between surfactant and co-surfactant was varied in order to obtain an optimal formulation of the nanoemulsion (CUN). Thereby, a formulation containing 4% cinnamon oil, 1% usnic acid, 37% surfactant: co-surfactant mixture and 58% deionized water as the continuous phase was used in further experiments, based on the lowest droplet size (96.39 ± 2.38 nm) and the highest percentage of transmittance (99.76%), among the investigated samples. In the liberation study of usnic acid from CUN, performed using Franz diffusion cells with a cellulose acetate membrane (acceptor medium being phosphate buffer solution of pH 5.5), a plateau of released usnic acid (93.19%) was reached after 8h of the experiment. CUN was stable over six months of observation, with a certain change in globule size, zeta potential and polydispersity index. Finally, CUB or CUN incorporated into hydrophilic gels prepared with Carbopol 980 and glycerol were used in an in vivo study investigating their topical application in a DMBA/croton oil-induced skin carcinogenesis in Swiss albino mice. It was shown that CUN-containing gel significantly reduced skin carcinogenesis in mice after 16 weeks of topical application, leading to the increase of animal body weight, decrease of tumor diameters and numbers, as well as to the reduction of tumor multiplicity and restoration of tumor yield. Also, topical application of CUN-containing gel significantly restored oxidative stress markers in animal tissues, namely, levels of SOD, CAT, GSH, GPx and total protein were restored after 16 weeks of the experiment. Such findings were attributed to the antitumor effects of both cinnamon oil and usnic acid reported in the literature, potentiated by their encapsulation into nanoemulsion.

## 3. Polymeric Nano- and Microcarriers

A number of polymer-based DDSs have been devised for delivering usnic acid [25,26,27,28,29]. However, this review will focus only on the polymeric nano- and microcarriers (i.e., micro and nanospheres (matrix and capsules), as well as nonspherical nanoparticles (nanofibers)) as systems developed with the aim of achieving maximum tissue compatibility and minimal cytotoxicity [13,30]. Thorough literature survey revealed attempts of usnic acid encapsulation into polymeric carriers of nano- and microsize with the aim of enhancing its antitumor and anti-inflammatory activity and/or reduction of hepatotoxicity. Fewer studies investigated loading of usnic acid into nonspherical nanoparticles (nanofibers) or microparticles for the improvement of antimicrobial activity, as seen in Table 3. Details of stated investigations are discussed in the next section.

### 3.1. Polymeric Nanocarriers

#### 3.1.1. Polymeric Nanoparticles

**Figure 4:**
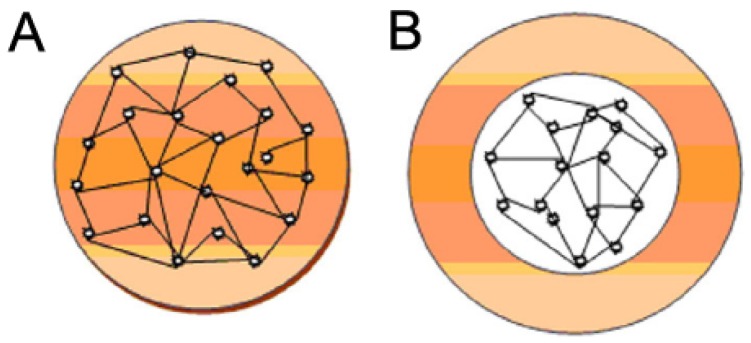
Schematic representation of polymeric nanoparticles: (**A**) nanospheres, (**B**) nanocapsules. Reprinted from Nanomedicine: Nanotechnology, Biology, and Medicine, 6, Mishra, B.; Patel, B.B.; Tiwari, S., Colloidal nanocarriers: a review on formulation technology, types and applications toward targeted drug delivery, 9–24 [30], Copyright (2010), with permission from Elsevier.

Polymer nanoparticles comprise nanospheres and nanocapsules (Figure 4). Nanospheres are matrix systems whose entire volume is filled with a polymer and the drug substance that is either dispersed or dissolved in this polymeric matrix or adsorbed on the surface of the nanosphere. On the other hand, nanocapsules represent a core–shell system composed of an oil or water core surrounded by a polymeric membrane, wherein the drug substrate can be found either in the core or the shell of the nanocapsule [13].

##### Polymeric Nanocapsules

In two studies [31,32], the great lipophilicity of usnic acid was circumvented by encapsulation into the oily cavity of polymeric nanocapsules as a strategy for improving its solubility, cellular uptake and antitumor efficacy. Therefore, nanocapsules were prepared with poly(lactide-*co*-glycolide) (PLGA 50:50), soybean oil as an oil core and soybean phosphatidylcholine and poloxamer F68 as surfactants, by interfacial deposition of a preformed polymer on the surface of oil-in-water emulsion, followed by lyophilization. Thus, PLGA nanocapsules (mean diameter = 324 ± 88 nm) containing usnic acid (1 mg/mL) were obtained with a drug: polymer ratio of 1:15 and a drug: oil ratio of 1:10. Usnic acid content (101.7 ± 1.7%) and encapsulation efficiency (99.4 ± 0.2%) were determined by HPLC, which was also used for the long-term stability study revealing the decrease of usnic acid content (77.0 ± 3.6%) after 36 months of storage at 4 °C. The in vitro release profile of usnic acid from PLGA nanocapsules, evaluated using the dialysis technique, revealed an initial burst of 15.1 ± 0.1% (attributed to the drug fraction adsorbed onto the particle surface), followed by the slow and gradual release reaching 78.30 ± 0.08% of the total content of usnic acid in the nanocapsules, leaving around 20% of the captured drug as a typical feature of these core–shell nanosystems.

The cytotoxicity of usnic acid assayed in vitro using MTT in NCI-H292 cells (mucoepidermoid lung cancer cells) suggested dose-dependent effects of both free and encapsulated usnic acid, revealing similar IC_50_ values (10.0 and 11.5 μg/mL, respectively). However, in further performed histological analyses, no changes in morphological characteristics of NCI-H292 cells were detected after treatment with encapsulated usnic acid compared to the free drug, especially when used in higher concentrations (5 μg/mL) [31]. The antitumor activity of usnic acid encapsulated in nanocapsules was further evaluated in vivo in male mice with Sarcoma-180 tumors. Animals were treated with a 0.5% suspension of usnic acid in Tween 80 or with usnic acid-loaded nanocapsules intraperitoneally once daily for 7 days at a dose of 15 mg/kg, whereby treatment of the animals was started 24 h after tumor inoculation. While untreated animals presented a progressive increase in tumor growth, the treatment with both free and encapsulated usnic acid produced a decrease in tumor mass. To this end, encapsulation of usnic acid was shown to increase its antitumor activity by 26.4% (compared to pure usnic acid). These findings were confirmed by further employed histopathological analysis of tumors. Namely, typical and atypical cells in constant mitosis were found after treatment with both forms of usnic acid. Nevertheless, more extensive necrotic areas with uncharacterized pyknotic nuclear cells in the tumor tissue were observed in animals treated with usnic acid-loaded nanocapsules. Accordingly, the mechanism of antitumor activity of usnic acid in Sarcoma 180-bearing mice was suggested to be necrosis without apoptosis, attributed to the inhibition of the mitochondrial function by disruption of the electron transport chain [31,32]. Hepatocytic necrosis was also revealed in histopathological analyses of the liver of examined mice with sarcoma, treated with both free and encapsulated usnic acid. However, vacuolization of hepatocytes and an intensive lymphocyte infiltration in portal spaces were noticed after treatment with free usnic acid. In contrast, only morphologically uncharacterized hepatocytes and a mild lymphocyte infiltration after treatment with usnic acid-loaded nanocapsules suggested significant reduction in the hepatotoxicity of usnic acid after encapsulation. Also, absence of renal and haematological toxicity as well as immunological effects were observed after treatment of Sarcoma 180-bearing mice with both free and encapsulated usnic acid [32].

##### Polymeric Nanospheres 

**Figure 5:**
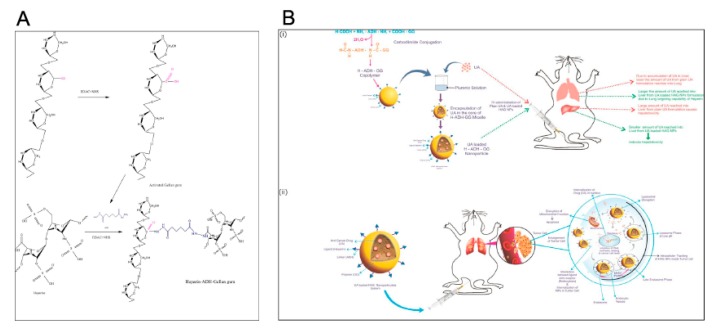
Synthetic scheme of (**A**) heparin-adipic acid dihydrazide copolymer (HAG) copolymer; (**B**) (i) proposed drug distribution phenomenon from plain UA and HAG nanoparticles and (ii) tumor targeting potential of prepared UA-loaded HAG nanoparticles. Reprinted from International Journal of Pharmaceutics, 557, Garg, A.; Garg, S.; Sahu, N.K.; Rani, S.; Gupta, U.; Yadav, A.K., Heparin appended ADH-anionic polysaccharide nanoparticles for site-specific delivery of usnic acid, 238–253 [33], Copyright (2019), with permission from Elsevier.

A novel study of Garg et al. [33] investigated usnic acid loaded into nanoparticles of gellan gum (GG) modified with heparin-adipic acid dihydrazide (ADH) copolymer (HAG), where heparin was used as a ligand that may bind to the receptor present in the tumor cells, with the aim of its site-specific delivery in the lung cancer cells (Figure 5). 

Nanoparticles, prepared using nanoprecipitation with Pluronic F-68 used as the surfactant followed by lyophilization, were investigated in a preformulation study, based on which a formulation containing drug: polymer ratio of 25:20 was chosen for further experiments. Prepared nanoparticles revealed high encapsulation efficiency (99.98% ± 0.50%), low particle size (83.38 ± 1.18nm) and PDI (0.074 ± 0.029) and negative zeta potential (ξ = −12.6 mV). Furthermore, FTIR and NMR spectroscopy analyses confirmed the presence of all materials used in the preparation of usnic loaded-nanoparticles, Atomic Force Microscopy (AFM) revealed their spherical shape, while differential scanning calorimetry (DSC) and powder X-ray diffraction suggested their crystalline nature. A liberation study showed a sustained release of usnic acid from the prepared nanoparticles (95.67% in 48 h). The in vitro cytotoxicity of usnic acid-loaded HAG nanoparticles against the A549 human lung cancer cell line was investigated by means of sulforodamine B (SRB) assay. Usnic acid loaded into nanoparticles was found to be cytotoxic in tested cancer cells to a greater extent with the concentration between 10 and 80 μg/mL, when compared to free usnic acid. Such a finding was correlated to the ligand-receptor binding proficiency of heparin and Anaplastic Lymphoma Kinase (ALK) overexpressed in the tested cells. In addition, it was suggested that heparin in aqueous solution helped to impart a “stealth” property of the nanoparticles, which was in line with the in vivo biodistribution assay performed in the albino rats that also revealed lower amounts of encapsulated usnic acid in the liver compared to the free drug. Cell cycle analysis indicated the antitumor mechanism of prepared usnic acid- loaded HAG nanoparticles in the A549 cells to be cell cycle arrest in the G2/M phase. Also, encapsulated usnic acid revealed lower hemolytic and haematological toxicity compared to the free drug. Cytotoxicity of usnic acid loaded in the HAG nanoparticles was justified by several additional in vitro assays performed in this study such as an apoptosis assay, DNA fragmentation assay, tumorsphere assay, cell cycle assay, and so forth, however, with no comparison to unloaded (free) usnic acid.

#### 3.1.2. Polymeric Non-Spherical Nanoparticles 

##### Polymeric Nanofibers

Another attempt to improve the antimicrobial activity of usnic acid as a bactericidal agent against *S. aureus* as a potential diabetic wound healing treatment was performed by its loading to polymeric Eudragit L-100 and polyvinylpyrrolidone (PVP) nanofibers [34]. Both nanofibers were prepared by introduction of polymer in ethanol (1.3 g of Eudragit L100 in 10mL of ethanol; PVP in 10 mL of ethanol in a ratio of 2:3 in mass), and after the addition of usnic acid (10 mg) the solutions were subjected to electrospinning. Thereby, concentrations of all components for the prepared solutions were defined according to adequate conditions for spinnability in an experimental setup and also in accordance to the MBC of usnic acid. SEM images of electrospun fibers of usnic acid-loaded Eudragit^®^ L100 matrix revealed the stranded-like fiber morphology, while in the case of usnic acid-loaded nanofiber prepared with PVP, negligible concentrations of beads and regular cylindrical structures could be observed, characterizing a regular and volumetric process of incorporating additives during production of fibers. Both usnic acid-loaded fibers significantly inhibited growth of *S. aureus* in contrast to the pristine polymeric fibers, assessed in the agar diffusion experiment, meaning that usnic acid released from the nanofibers was responsible for the observed antimicrobial effect. Aiming to establish a connection between the kinetics of usnic acid release and inhibition of growth of investigated bacteria, an in vitro liberation study was further performed. It was shown that a typical diffusion of usnic acid along fiber walls dictated the kinetics of the overall release. Also, improved release of usnic acid after 10 min of the experiment was provided by nanofibers prepared with Eudragit^®^ L100 (0.045 mg/mL) in comparison to the PVP matrix (0.040 mg/mL of usnic acid; both concentrations being around the MBC of usnic acid against *S. aureus*). In addition, it was shown that release of usnic acid from the Eudragit^®^ L100 matrix was pH-activated. These findings indicated combined action of both parameters (usnic acid concentration and pH of release media) to regulate the kinetics of bactericidal action. Therefore, in the opinion of the authors, the developed usnic acid-loaded nanofibers might represent an important advance in the direction of development of new antibacterial materials with pH-controlled release.

### 3.2. Polymeric Microspheres

Usnic acid was incorporated into PLGA microspheres for the first time in the study of Ribeiro-Costa et al. [35] in an attempt to maximize the therapeutic activity while reducing side effects of this proven anticancer drug. Based on the preformulation study, PLGA microspheres (drug:polymer = 1:45) were prepared by the double emulsion method with the addition of poly ethylene glycol (PEG) and polyvinyl alcohol (PVA) as a stabilizer, followed by lyophilization. This enabled production of microspheres having sizes of 7.02 ± 2.74 μm with high entrapment efficiency (around 100%) and acceptable levels of usnic acid content (90%) within 7 months of storage at 4 °C. The in vitro release of usnic acid from PLGA-microspheres revealed a typical bimodal pattern (initial burst effect, followed by a gradual release of usnic acid reaching 92% ± 0.04% within 5 days). The encapsulation of usnic acid compared to its free form did not cause a significant difference in an in vitro cytotoxicity assay in the Larynx epidermoid carcinoma cell line (HEp-2), while IC_50_ values of unloaded and microsphere-loaded usnic acid were of the same order of magnitude. However, intraperitoneal administration of the encapsulated usnic acid promoted an increase in tumor inhibition activity (21% compared with the free usnic acid) in mice against Sarcoma-180 tumors. In addition, histopathological analysis of tumors and livers of the sacrificed animals revealed extensive necrosis after treatment with free, but not encapsulated usnic acid. Also, an in vivo investigation of Marihno et al. [40] showed that encapsulation of usnic acid into PLGA microspheres minimized its hepatotoxic potential during pregnancy. Comparing the results of the study of Ribeiro-Costa et al. (2004) [35] to the previously discussed inferences on PLGA nanocapsules loaded with usnic acid [32], while taking into account the same experimental setup in both studies, it can be concluded that encapsulation into nanocarriers may be considered more effective than encapsulation into microcarriers in the enhancement of antitumor activity of this drug. Such a finding might be related to the faster penetration of nanocapsules (<200 nm) than the microspheres (7 μm) into the tumor tissue, emphasizing the importance of particle size on the bioavailability of usnic acid. 

In a novel study of Barbosa et al. [36], usnic acid was encapsulated into poly-𝜀-caprolactone (PCL) microspheres using the multiple emulsion method following solvent evaporation. PCL microspheres were characterized by the encapsulation efficiency (97.72%), particle size, polydispersity (span), and zeta potential (ξ) (13.54, 2.36 and −44.5 ± 2.95 μm for usnic acid-loaded and 9.37, 2.18 and −26.9 ± 0.58 μm for “empty” microspheres, respectively). Barbosa et al. compared the anti-inflammatory activity of usnic acid incorporated in PCL microspheres and free usnic acid, applying in vivo assays (the subcutaneous air pouch and carrageenan-induced paw edema in rats) and measuring the inflammatory cytokines and neutrophil granule myeloperoxidase (MPO) levels, the inflammatory mediators secreted after stimulation of polymorphonuclear leukocytes released into injured tissue. The authors suggested the mechanism of the shown anti-inflammatory activity to be due to registered attenuated levels of MPO (involved in reactive oxygen production, free radicals, and membrane oxidation, commonly related to bacterial lysis and tissue oxidative injury). In addition, the obtained results suggested that the anti-inflammatory effect of free usnic acid and usnic acid incorporated into microspheres was a consequence of the negative regulation of iNOS, IL-1𝛽 and TNF-𝛼 (related to increase of vascular permeability and migration of cells that produce chemokines), expressed by a significant decrease in the leukocytes migration and TNF-𝛼, IL-1𝛽 and NO levels in the inflammatory exudate from subcutaneous air pouch. Although the results indicated significant anti-inflammatory activity in the case of both usnic acid forms investigated, a greater effect was observed when usnic acid was incorporated into PCL microspheres. In this study, acute toxicity was accessed in vivo in mice receiving usnic acid either in free (2000, 300 and 50mg/kg; p.o.) or PCL encapsulated form (2000 mg/kg; p.o.), compared to indomethacin (10 mg/kg; p.o.) as positive control, or vehicle (0.9% NaCl: 5% Cremophor; p.o.) as negative control. It was shown that animals treated with usnic acid in the highest tested dose (2000 mg/kg) presented clinical symptoms of toxicity and death, while in the dose of 300 mg/kg, only reduced food intake and weight loss were observed. However, no indication of acute toxicity or occurrence of death was observed in the groups treated with free usnic acid in the lowest dose (50 mg/kg; p.o.) or usnic acid encapsulated into PCL microspheres. Further histopathological analyses of kidneys of sacrificed animals submitted to acute toxicity investigation exhibited serious liver and kidney damage after treatment with usnic acid in the highest dose (2000mg/kg), i.e., morphological changes with some damage in liver/kidney tissue in animals treated with free (at dose of 300 and 50 mg/kg) and encapsulated usnic acid. It was concluded that usnic acid showed equivalent or greater anti-inflammatory activity after being incorporated into PCL microspheres compared to free usnic acid, with greatly reduced acute toxicity.

Recently, Martinelli et al. [37] synthesized carboxylated poly(l-lactide)s (CPLLAs) by polymerizing l-lactide and 2,2-bis(hydro-methyl)propionic acid, with the aim of introducing a polar head on the hydrophobic polymer chain, providing a surfactant-like structure to avoid usage of surfactants in the fabrication of microparticles, intended for the local eradication of microbial biofilms with possible usage in wound dressings. Since CPLLA with a molecular weight of 3300 g/mol exhibited optimal surfactant-like behavior, this polymer was used to make microparticles with usnic acid, prepared by the emulsion–evaporation method, with a high encapsulation efficiency (80%, corresponding to 80 μg of usnic acid per microparticle). Loading of the microparticles with usnic acid induced the decrease of its mean particle diameter (2.4 ± 0.8 and 1.4 ± 0.5 μm for the unloaded and loaded microparticles, respectively), which was related to the hydrophobic interactions between the polymer and usnic acid, confirmed by DSC. The release of usnic acid from CPLLA microparticles was examined in phosphate buffer (pH 7.4) at four different temperatures, i.e., at 4, 25, 37 and 42 °C, investigated as the storage, room, normal and increased body temperature (in the case of wound infections), respectively. The release of usnic acid from CPLLA microspheres was found to be bimodal (initial burst effect, followed by a slower release up to 80 h) at all investigated temperatures, while the percentage of the released drug expectedly increased with increasing temperature. Evaluation of the kinetic parameters indicated liberation of usnic acid to be driven by its diffusion through the amorphous regions of the polymer. Thereby, the bimodal release behavior was related to the different structures of usnic acid in the system, i.e., its amorphous and crystalline form enabled rapid and slower release, respectively. Further testing of the antimicrobial activity of free and usnic acid loaded into CPLLA microspheres against *Staphylococcus epidermidis* both in planktonic and biofilm states was assessed in vitro. Thereby, MIC values obtained using the broth microdilution assay for free usnic acid were found to be lower compared to CPLLA microparticles loaded with usnic acid against tested strain of *S. epidermidis*. On the other hand, for the antibiofilm activity assessment, 24 h old *S. epidermidis* biofilms were challenged with unloaded and usnic acid-loaded CPLLA microparticles *versus* free usnic acid in suspension. The number of detached CFU s/mm^2^ of glass coverslide surface after each treatment indicated no antibiofilm activity of the unloaded microparticles. Also, better ability of the encapsulated usnic acid to kill bacteria in the biofilm compared to free usnic acid used in the same concentration was revealed and attributed to previously observed controlled release of usnic acid from the microparticles. 

In a novel approach [38], microparticles prepared from polylactic acid (PLA) and PVA were used as the carriers of usnic acid. Afterwards, these microparticles were deposited in the form of thin coating to titanium (Ti) substrate, by means of matrix-assisted pulsed laser evaporation (MAPLE). Ti substrate was chosen for this research as a material routinely used for implants in bone fractures and dental work, while recent studies suggested frequent colonization of these implants by biofilm-developing bacteria. Also, MAPLE was selected as the laser technique based on a cryogenic approach for transferring polymeric materials onto a substrate in a “preserved” manner (in this scenario in a microparticulate form, preferably providing controlled release of usnic acid). Before deposition to Ti, microspheres were prepared using an emulsion–evaporation method. The optimum deposition laser fluence in MAPLE identified using a comparative IR mapping analysis allowed fabrication of microsphere-based thin coatings with average diameters of 5 µm (the same as in microsphere suspension), as revealed by SEM. Biocompatibility of the developed micro-biocoatings was assessed in mesenchymal stem cells (MSCs) by means of microscopic analysis after seeding the cells on the investigated Ti substrates. Normal development (morphology and growth) of MSC cells on both uncoated and coated slides was shown, demonstrating that the microsphere-based coatings had neither cytotoxic nor inhibitory effects. Formation of *S. aureus* biofilm on uncoated and coated Ti substrates was analyzed using a static model for monospecific biofilm development. Viable cell counts (VCCs) of microorganisms grown in biofilms were performed after 24 h to evaluate the initialization of biofilm formation, i.e., after 48 and 72 h, for mature biofilm formation. It was demonstrated that the developed micro-biocoatings possessed great antibiofilm effects against *S. aureus*, while both initial attachment of *S. aureus* and biofilm formation (after 24 h) as well as the biofilm maturation (after 48 and 72 h) were altered in the coated Ti substrate, compared to the control (uncoated Ti substrate).

In a similar study, the MAPLE technique was used for the deposition of thin coatings containing magnetic PLGA-PVA microspheres loaded with usnic acid to the Ti substrate, for the design of new surfaces resistant to *S. aureus* colonization [39]. Thus, magnetite nanoparticles (designated Fe_3_O_4_@C_14_) were synthesized by precipitation of Fe^2+^ and Fe^3+^ into basic aqueous solutions of myristic acid. The prepared magnetic nanoparticles with a hydrophobic, nonpolar shell represented by a C_14_ alkyl chain were mixed with the hydrophobic polymer (PLGA) and usnic acid in chloroform. In the second stage, the hydrophilic polymer (PVA) water solution was added to the hydrophobic mixture, and an amphiphilic system (marked as PLGA-PVA-Fe_3_O_4_@C_14_-UA) was obtained using a solvent evaporation method. The presence of Fe_3_O_4_@C_14_ nanoparticles (spherical in shape, with a diameter of 5-20 nm) in the PLGA-PVA-Fe_3_O_4_@C_14_-UA microspheres (having dimeter of 1–20 µm) was confirmed by TEM and selected area electron diffraction (SAED) analyses. Similar shapes and morphologies were identified after Ti substrates were coated with PLGA-PVA-Fe_3_O_4_@C_14_-UA microspheres, proving that their structure was not affected by MAPLE deposition. The quantitative evaluation of MIC values of the of PLGA-PVA-Fe_3_O_4_@C_14_-UA, assessed using a broth microdilution assay, suggested these microspheres to inhibit the planktonic cell growth of *S. aureus* in a dose-dependent manner. In was of further interest to investigate whether the revealed antimicrobial activity of PLGA-PVA-Fe_3_O_4_@C_14_-UA was preserved after being used as thin films obtained by MAPLE. Therefore, formation of *S. aureus* biofilms on uncoated and coated Ti substrate was analyzed using a static model for monospecific biofilm development. As in a previously described study of the same research group [38], VCCs were measured after 24, 48, and 72 h. It was demonstrated that the developed microspheres inhibited both the initial attachment of *S. aureus* to the coated surfaces (lower VCCs compared to the control, after 24 h) and the development of mature biofilms (lower VCCs compared to the control after 48 and 72 h). Also, in vitro bioevalution tests performed on the fabricated thin films revealed their great biocompatibility, assessed in MSC cells.

## 4. Nonorganic Nanocarriers

### 4.1. Magnetic Nanoparticles

Magnetic nanoparticles have been intensively investigated in recent years as carriers for substances having limited therapeutic usage connected to their toxicity or unfavorable physico-chemical properties. Due to the possible magnetic field-induced aggregation, surface oxidation and lack of functional groups, as major drawbacks of magnetic nanoparticles, their surface is often modified (i.e., coated with polymers, surfactants or inorganic materials), resulting in core–shell structures that may enhance their water solubility, provide protection against oxidation and increase drug entrapment efficiency [41].

In a series of studies, a research group of Grumezescu and colleagues investigated nanostructures based on magnetite (Fe_3_O_4_) as carriers of usnic acid [42,43,44,45]. These investigations, based on the ability of Fe_3_O_4_ to inhibit microbial film development, represented an attempt to exploit this intrinsic property of magnetite, alongside the well-known antimicrobial activity of usnic acid, thus yielding nanoparticles with potential use in medical device-related infections and/or wounds and burns treatment, as conditions usually highly resistant to therapy based on conventional antibiotics.

In one of their earlier studies [42], uncoated magnetite nanoparticles were prepared with usnic acid by nanoprecipitation (designated Fe_3_O_4_@UA). The mean diameter of the usnic acid-loaded nanoparticles was 10 nm, with usnic acid content of around 6.4%. MIC values of Fe_3_O_4_@UA, assessed using a broth microdilution assay, revealed a significantly improved inhibition of planktonic cell growths of *E. faecalis* and *Escherichia coli* compared with the control (Fe_3_O_4_). In addition, usnic acid-loaded Fe_3_O_4_ nanoparticles exhibited a significant inhibitory effect on the biofilm formed by Gram-positive bacteria (*S. aureus* and *E. faecalis*), in contrast to the Gram-negative strains where they inhibited *E. coli* biofilm development only at high concentrations, while in biofilms of *Pseudomonas aeruginosa* no inhibitory effect was observed at all. In another study [43], oleic acid was used for coating of Fe_3_O_4_ nanoparticles (designated FeOA) using the Massart method in microwave conditions, followed by adsorption of usnic acid (designated FeOAU). The developed core–shell nanocomposite was coated onto coverslips, and their antibiofilm effect in *S. aureus* biofilm formation was assessed in comparison to the coverslips coated with FeOA (negative control) and with usnic acid (positive control). FeOA-coated coverslips showed no antibiofilm activity, while FeOAU-coated coverslips exhibited an improved antibiofilm effect in *S. aureus* biofilm formation, revealed by a drastic decrease of the VCCs (quantitative assay), as well as qualitatively (by means of confocal laser scanning microscopy), compared to usnic acid pelliculized on the coverslips (positive control). However, it was later reported that there were some limitations of the obtained nanocomposite, probably related to the apolar nature of the used organic shell of oleic acid, making them insoluble in water, thus promoting their aggregation in aqueous solution [42].

Uncoated Fe_3_O_4_ nanoparticles developed by Grumezescu et al. [42] have been used in further studies of this research group aiming at preparing novel wound dressings with improved antimicrobial properties by combination of the biodegradability properties of polymers (sodium alginate, AlgNa, carboximethylcellulose, CMC [44] and PVA [45]) with the antimicrobial and antibiofilm properties of usnic acid, potentiated by the magnetite nanoparticle carrier. Thereby, thin films based on polymers and usnic acid-loaded Fe_3_O_4_ nanoparticles were prepared using the solvent casting method, followed by freeze-drying. Further assessed SEM micrographs of the prepared films revealed an open, interlaced and highly porous network with homogeneously dispersed nanoparticles (10 nm in size) in the wound dressing matrix prepared with AlgNa (designated AlgNa/Fe_3_O_4_@UA), i.e., nanoparticle aggregates on the surface of wound dressings prepared with CMC (designated CMC/Fe_3_O_4_@UA) [44]. In the case of usnic acid-loaded magnetite nanoparticles incorporated into wound dressing matrix prepared with PVA (designated PVP-Fe_3_O_4_@UA), SEM analysis revealed a smooth surface with a few cavities [45]. Biocompatibility assays showed no cytotoxic reactions of any of the developed wound dressings. Microbiological testing of all fabricated wound dressings was assessed in an adopted disk diffusion method. Thereby, wound dressings were added to the inoculated plates at three time points: immediately after *S. aureus* inoculation and after 6 and 12 h, after which the plates were further incubated for 24 h at 37 °C prior to inhibition zone readings. In a study of Grumezescu et al. [44], AlgNa/Fe_3_O_4_@UA was demonstrated to induce more effective bacterial growth inhibition compared to CMC/Fe_3_O_4_@UA. Observed microbicidal effects of AlgNa/Fe_3_O_4_@UA on microbial cultures of different densities (in all time points) was observed and was attributed to Fe_3_O_4_@UA activity. It should be noted that the antimicrobial activity decreased as the microbe density increased (i.e., later time of wound dressing addition to bacterial inoculum). Comparable results were obtained for usnic acid-loaded magnetite nanoparticles incorporated into a wound dressing matrix prepared with PVA (PVP-Fe_3_O_4_@UA), which exhibited great antimicrobial potential considering the observed significant inhibition of *S. aureus* development, even in the presence of high bacterial densities [45].

Taresco et al. [41] investigated functionalized core–shell manganese iron oxide nanoparticles (MnFe_2_O_4_) as delivery systems for usnic acid, potentially used for the prevention and treatment of medical device-related infections. MnFe_2_O_4_ was chosen due to its strong magnetism and safety for humans, while the surface properties (hydrophilicity/hydrophobicity, functional groups) were modified using two polymers for their coating: (1) a hydrophilic cationic polyacrylamide bearing tertiary amino groups (pAcDED; due to the intrinsic antimicrobial activity, basic groups for usnic acid adsorption and the possibility of binding to the negative bacterial cell membrane), and (2) a newly synthesized star-branched polycaprolactone (sbPCL_50_; due to expected hydrophobic interactions with usnic acid). MnFe_2_O_4_ nanoparticles were synthesized by co-precipitation of Fe^3+^ and Mn^2+^ from water-in-toluene microemulsion. Further coating with sbPCL_50_ was performed by physical adsorption of the polymer onto the surface of magnetic nanoparticles yielding a sample designated sbPCL_50_-MnFe_2_O_4_. Nanoparticle coating with pAcDED was carried out using two methods: (1) physical adsorption (same procedure as in the case of sbPCL_50_), designated as pAcDED-MnFe_2_O_4_ and (2) in situ polymerization and cross-linking of the monomer AcDED (previously adsorbed onto nanoparticles) with N,N methylenebisacrylamide, designated as pAcDED_CL_-MnFe_2_O_4_. For drug encapsulation, the uncoated and coated nanoparticles were kept in contact with an alkaline solution of usnic acid. TEM analysis indicated a high degree of aggregation of uncoated nanoparticles, which was greatly reduced by polymeric coating. Although some degree of aggregation could also be observed in polymer-coated samples, it was possible to distinguish individual inorganic nuclei from 8 to 11 nm in diameter. Zeta potential analyses revealed negative charges of uncoated nanoparticles (−21mV), due to the presence of OH groups on their surface, which were significantly changed after coating with sbPCL_50_ (+1 mV) and pAcDED-MnFe_2_O_4_ (+20 mV). Encapsulation efficiency of usnic acid was also influenced by polymeric coating, with the highest amount being incorporated in cationic polymer-coated nanoparticles (0.48 ± 0.05% and 0.62 ± 0.02% for pAcDED_CL_-MnFe_2_O_4_ and pAcDED-MnFe_2_O_4_, respectively, compared to 0.22 ± 0.04% in the uncoated and 0.49 ± 0.07% for sbPCL_50_-coated nanoparticles). The lower amount of adsorbed usnic acid in pAcDED_CL_-MnFe_2_O_4_ compared to pAcDED-MnFe_2_O_4_ was probably related to the lower ability of the cross-linked polymer to swell, resulting in adsorption of usnic acid mainly on the surface of nanoparticles. As expected, the type of interactions between usnic acid and the polymers significantly affected its release from the nanoparticles. Although a plateau was reached in all nanoparticles after 24 h of the experiment, pAcDED_CL_-MnFe_2_O_4_ released the highest percentage of usnic acid (more than 90%), confirming its adsorption mainly on the surface of the polymeric shell. A slightly lower percentage of released usnic acid (about 65%) was observed from the pAcDED-MnFe_2_O_4_ sample, probably due to the diffusion controlled by the polymeric coating and the acid–base interaction of usnic acid and polymer. Finally, uncoated nanoparticles (MnFe_2_O_4_) and those coated with the hydrophobic polymer sbPCL_50_-MnFe_2_O_4_ showed lower release of usnic acid (about 40%), suggesting a good affinity of the drug to the hydrophobic nanoparticles. Antimicrobial activity was assessed using two methods against *Staphylococcus epidermidis*. Disc diffusion showed the highest percentage of inhibition in uncoated nanoparticles, followed by hydrophobic polymer-coated nanoparticles (sbPCL_50_-MnFe_2_O_4_), while the lowest inhibition percentage was observed in hydrophilic polymer-coated nanoparticles (pAcDED_CL_-MnFe_2_O_4_ and pAcDED-MnFe_2_O_4_, respectively). Such results were explained by the dependence of the interaction of usnic acid and the magnetite nanoparticles, i.e., higher inhibition zone was explained by weaker usnic acid–nanoparticle interaction, as opposed to the findings of the liberation study. On the other hand, the broth microdilution method showed that all the tested samples inhibited the growth of *S. epidermidis* in a dose-dependent manner, with MIC values being significantly lower for the nanoparticles coated with hydrophilic polymer (pAcDED_CL_-MnFe_2_O_4_ and pAcDED-MnFe_2_O_4_) than the ones obtained for the uncoated nanoparticles and nanoparticles coated with a hydrophobic polymer (sbPCL_50_-MnFe_2_O_4_), which was explained by the antimicrobial activity of the polymer itself. 

**Figure 6:**
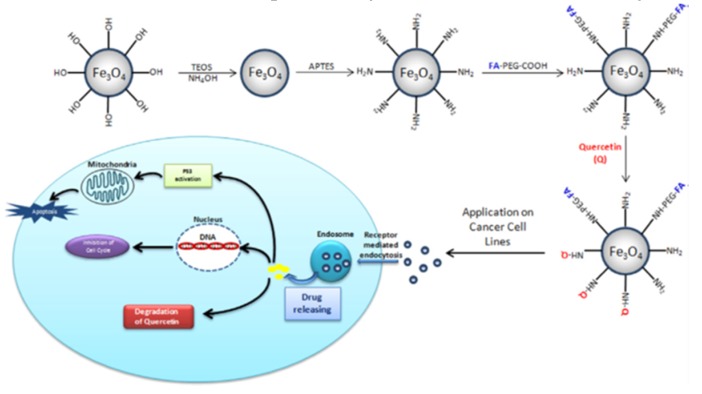
Schematic illustration of synthesis of superparamagnetic iron oxide nanoparticles (SPION) functionalized with (3-Aminopropyl)triethoxysilane (APTES), after which carboxylated polyethylene glycol (PEG-COOH), folic acid (FA) and carboxylate quercetin (CQ) were conjugated on the surface via a carboxylic/amine group using the nanoprecipitation method (designated SPION@APTES@FA PEG@CQ) and its effect mechanism on cells (in a study of Alpsoy et al. [46], CQ was replaced by usnic acid). Reprinted from Ceramics International, 42, Akal, Z.U.; Alpsoy, L.; Baykal, A., Heparin appended ADH-anionic polysaccharide nanoparticles for site-specific delivery of usnic acid, 9065–9072 [47], Copyright (2016), with permission from Elsevier.

In another study [46], Fe_3_O_4_ nanoparticles were functionalized with (3-Aminopropyl)triethoxysilane (APTES) using the Stöber method, after which carboxylated polyethylene glycol (PEG-COOH), folic acid (FA) and usnic acid were conjugated on the surface via a carboxylic/amine group using the nanoprecipitation method (Figure 6). These resultant nanoparticles were investigated as potential carriers for in vitro analysis of cytotoxicity against several cancer cell lines. Obtained nanoparticles had the size of 20 nm (revealed by TEM). X-ray powder diffraction confirmed the single-phase crystal formation, while FTIR analyses explained the conjugation of all functional groups to the surface of nanoparticles. Usnic acid liberation studies assessed in vitro showed its enhanced release from the nanoparticles at pH 5.4 compared to pH 7.4 after 72 h, which might be considered a favorable release profile considering a more acidic environment of cancer cells than the healthy ones. Such a result was explained by deprotonation of amine group linkage between silica and usnic acid molecules, which weakened this bond and increased usnic acid release. Also, cytotoxicity results of prepared nanoparticles, assessed by means of MTT assay, indicated its increased activity against L929 (mouse fibroblast cell line) and A549 (human lung cancer cell line) cancer cells as compared to other cancer cells (U87 (glioblastoma cell line, brain cancer), HeLa (cervix cancer cell line) and MCF-7 (breast cancer cell line)).

### 4.2. Diamond Nanoparticles 

**Figure 7:**
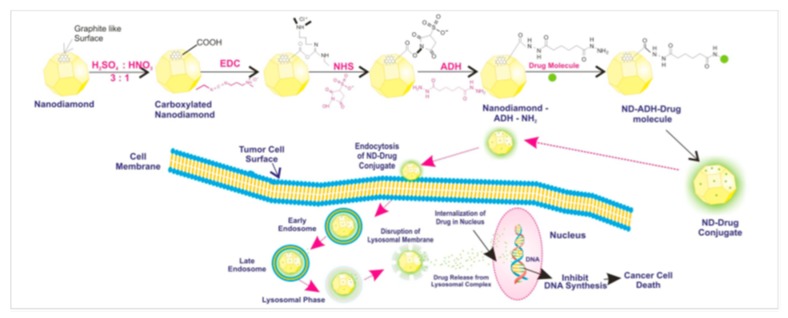
Graphical representation of conjugation pattern of ND–drug conjugates and the proposed hypothesis of the tumor targeting strategy of the ND–drug conjugate. Reprinted from Diamond and Related Materials, 94, Garg, S.; Garg, A.; Sahu, N.K.; Yadav, A.K. Synthesis and characterization of nanodiamond-anticancer drug conjugates for tumor targeting, 172–185 [48], Copyright (2019), with permission from Elsevier.

Nano diamonds (ND) are getting increasing scientific attention as DDSs, as they possess low toxicity, high chemical stability, high affinity for biomolecules, and they are easily surface-functionalized [48]. In a recent study, three anticancer drugs—usnic acid (UA), 5-fluorouracil (5-FU) and curcumin (CUR)—were introduced to ND previously chemically modified using N-(3-dimethylaminopropyl)-N′-ethylcarbodiimide hydrochloride (EDC-HCl), N-hydroxysuccinimide (NHS) and ADH, as shown in Figure 7. 

The resultant ND–drug conjugates were designated ND-ADH-UA, ND-ADH-5-FU and ND-ADH-CUR, respectively. Spectroscopic analyses (NMR, ESI-MS and FTIR) confirmed the conjugation of ND with all the investigated anticancer drugs. AFM and SEM photomicrographs revealed that all ND-drug conjugates displayed flake-like structures with preferred crystal orientation, while DSC curves indicated the presence of all anticancer drugs in crystalline nature. ND–drug conjugates were observed to have diameters of 114.32, 81.6 and 39.85 nm, i.e., loading efficiencies of 85.3 ± 0.25%, 88.14 ± 1.38% and 93.8 ± 0.85% for ND-ADH-UA, ND-ADH-5-FU and ND-ADH-CUR, respectively. The negative values of the zeta potential analyses of ND conjugates (−10.6, −11.2 and −14.3 mV for ND-ADH-UA, ND-ADH-5-FU and ND-ADH-CUR, respectively) were explained by the presence of the carboxyl moiety in the ND. The release of the drugs from ND–drug conjugates was examined at 37 °C as normal body temperature, at different pH values, in order to analyze their feasibility as DDSs for anticancer drugs (pH 7.4, as physiological pH value; pH 5.5, mimicking acidic endosome environment; and pH 6.5, simulating tumor environment). It was shown that all ND–drug conjugates offered sustained release explained by the presence of an amide bond between the drug and ND, which is relatively more stable than physical absorption, leading to slower release of the anticancer drugs. Release of the investigated drugs from the ND–drug conjugates was also pH-dependent, i.e., enhanced with lowering the pH value, which was ascribed to the cleavage of amide bond in the acidic environment. Although the SRB assay assessed in vitro revealed dose-dependent activity of both ND–drug conjugates and their free counterparts in two cancer cell lines (MCF-7, breast cancer and Hep-G2, human hepatoma), the comparison of the observed cytotoxic effects was not discussed by the authors. However, lower hemolytic toxicity of all ND–drug conjugates, assessed in an in vitro test using human blood, was shown in comparison to the appropriate free drugs. Results from docking and pharmacophore mapping analyses suggested all ND–drug conjugates to possess effective interactions with significant proteins, and the results obtained from pharmacophore mapping specified that the prepared ND–drug conjugates were specifically matched with the hypo 1 model with good fit values. 

## 5. Stability of Usnic Acid-Loaded Nano- and Microcarriers

During the development of new drug formulations, including the ones based on nano- and microcarriers, it is necessary to perform adequate stability studies that should include the testing of all parameters susceptible to change during transportation and storage that are likely to influence the safety, efficacy and quality of these products. However, in the presented papers dealing with encapsulation of usnic acid into nano- and microcarriers, only a few of them conducted the investigations on physico-chemical stability of the developed formulations, i.e., liposomes [15,17,19,20], nanoemulsion [24], polymeric nanocapsules [31] or microspheres [35]. 

When it comes to the methodology used in the stated studies, three studies [15,16,31] included assessment of selected parameters at defined storage conditions and defined time points in both accelerated and long-term conditions, whereby in the accelerated stability, investigation formulations were subjected to (1) centrifugation, (2) horizontal mechanical stirring, and (3) freeze–thaw cycles. For instance, in the investigation of Lira et al. [15], accelerated stability studies were used in the preformulation phase with the purpose of selecting optimal formulation with the highest amount of encapsulated usnic acid and concomitant good physico-chemical stability. Therefore, several batches of liposomes were prepared with soya phosphatidylcholine and cholesterol (neutral), with the addition of stearylamine or phosphatidic acid for positively and negatively charged liposomes, respectively. It was shown that positively charged liposomes composed of 42 μmol of lipids/10 μL of buffer and 1.5 mg/mL of usnic acid (drug:lipid ratio = 1:12) preserved their stability after accelerated stability testing without significant changes to their initial characteristics (particle size and zeta potential). In contrast, neutral usnic acid-loaded liposomes were completely unstable 2 h after preparation, shown by drug precipitation, while negatively charged liposomes exhibited precipitation after centrifugation. For long-term stability evaluation, positively charged liposomes were lyophilized, after which they were stable for 24 months (Table 2). As previously discussed, great encapsulation efficiency and stability of positively charged liposomes were attributed to unipolar interactions associated with the hydrophobic forces of this molecule and phosphatidylcholine bilayers, but also the presence of a positively charged lipid in the bilayer (stearylamine). Thus, in their further study, Lira et al. [17] prepared positively charged liposomes using the same lipids (soya phosphatidylcholine, cholesterol and stearylamine) entrapping usnic acid complex with β-cyclodextrin. In the accelerated stability study, the obtained liposomal suspension with 1.2 mg/mL (12:1 lipid:drug molar ratio, i.e., 42 mM of lipids), stored at 4 °C, preserved initial properties (milky appearance with a typically bluish reflection) after being submitted to centrifugation. Also, the formulation remained stable after 27 freeze–thaw cycles, while mechanical stress lead to redispersible creaming after 48 h. In addition, a progressive decrease of pH value of liposomal suspension was observed after 2 months (from 7.4 to 6.4). Therefore, in order to increase stability of prepared liposomes, they were lyophilized, and in this form, formulation was stable for five months (Table 2), with usnic acid content remaining unchanged during the time of investigation (around 100%). Similar to Lira et al. [15], in the work of Santos et al. [31], PLGA nanocapsules loaded with usnic acid in the form of suspension were subjected to an accelerated stability study in the preformulation phase by evaluating the macroscopic appearance of the prepared formulations, as well as measuring the particle size and pH value. Based on these results, formulation in which the highest usnic acid entrapping was achieved while preserving its good physico-chemical stability was chosen (1 mg/mL, drug: polymer ratio of 1:15 and a drug: oil ratio of 1:10). In a further long-term stability study, this formulation revealed a gradual decrease of pH (from 7.40 ± 0.03 to 6.98 ± 0.05) after 120 days of storage at 4 °C, suggesting its instability. However, after lyophilization, this formulation was stable for 36 months keeping initial macroscopic appearance and particle size diameter.

In another study investigating lyophilized PLGA microparticles [35], solely their long-term stability was investigated by monitoring macroscopic and microscopic appearance, pH value of the solution after redispersion and usnic acid content in predetermined time points (7–510 days of storage at 4 °C). Acceptable levels of usnic acid content (90% compared to the initial value of 105 ± 5.07%) was maintained for 7 months (Table 3), followed by its decrease reaching 61 ± 6.7% at the end of the experiment. In two novel studies, the surface of liposomes was functionalized with glycosidic moieties [19] or N-oxide/quaternary ammonium moiety [20] in order to enhance biological effects (antimicrobial and antioxidative, respectively) of the encapsulated usnic acid. The evaluation of these systems’ stabilities was achieved by monitoring particle sizes of the prepared formulations and their distribution (assessed using dynamic laser light scattering (DLS) measurements) and revealed their poor stability with notable particle aggregation within only two weeks. Similarly, in an investigation of the nanoemulsions containing usnic acid dispersed in the cinnamon oil [24], stability was assessed by measurements of particle size, PDI and zeta potential. It was shown that the prepared nanoemulsion was stable after 180 days of storage, without significant changes in the assessed parameters (particle size, PDI and zeta potential being 103.39 nm, 0.257% and −24.05 mV, respectively, compared to the initial values of 96.39 nm, 0.250% and −27.13 mV).

## 6. Concluding Remarks

This comprehensive literature survey on usnic acid loaded into nano- and microcarriers clearly points out this approach to have beneficial effects on the enhancement of physico-chemical properties, biological activities and/or reduction of toxicity of this lichen secondary metabolite.

Overall, when considering investigations of biological activities of usnic acid after encapsulation into nano- and microcarriers reviewed in this paper, antimicrobial activity was assessed only using in vitro tests. The obtained results were difficult to compare, even if the same type of formulations were investigated, due to the various types of antimicrobial tests performed. Additional concerns regarding the in vitro antimicrobial testing might arise from the fact that the investigated nano- and microsized DDSs consist of many components that may evince effects against bacteria (in both sessile and planktonic form) if the experimental setup is not adequately preset. This also applies to the in vitro antioxidant test, as well as cytotoxicity investigations, which were used either for the purpose of assessment of anticancer activity or biocompatibility, as a prerequisite of safe delivery of the tested nano- and microcarriers. Only a few studies investigating usnic acid encapsulation into polymeric nanocapsules and microspheres included assessment of biocompatibility in vivo, alongside investigations of its antitumor/anti-inflammatory activity. Conversely, some in vivo studies, such as research on wound healing of usnic acid loaded into liposomes incorporated into polymeric films or nanoemulsion prepared with usnic acid and cinnamon oil suggesting the significant reduction of skin carcinogenesis, failed to provide data concerning the safety aspects of these preparations. In addition, it should be emphasized that most of the investigations covered by this review did not include stability testing of the developed nano- and microsized formulations, opening questions of their quality, safety and efficiency. 

Stated reports have described recent advances that might resolve the existing disproportion between the great pharmacological potential of this compound on the one side, and its current scarce therapeutical application on the other. Nevertheless, additional comprehensive studies providing thorough information on physico-chemical and biopharmaceutical properties of these nano- and micronized DDSs, as well as their safety and efficiency, both in experimental and clinical setup, are needed before their practical usage in the treatment of inflammatory and oxidative stress-mediated diseases, including wounds and cancer, and bacterial infections caused by strains resistant to currently used antibiotics. 

## Figures and Tables

**Table 1:** List of products containing usnic acid as an active ingredient [11,12].

Product Name	Producer	Active Ingredients	Utilization
Micofoot Zeta	Zeta, Italy	Undecenoic acid, usnic acid, salicylic acid, aluminum acetate	Disinfection of skin, burns, and wounds; fungal skin infections
Foot Zeta	Zeta, Italy	Undecylenic Acid, usnic acid	Burns; fungal skin infections; wounds.
Steril Zeta	Zeta, Italy	Cream: Triclosan, usnic acid, hamamelis, cod-liver oil	Disinfection of burns and wounds
		Topical powder: Triclosan, usnic acid, zinc oxide, zinc stearate	
Scabicid	Scabicid Kimia, Indonesia	Lindane, usnic acid	Gram-positive bacterial skin infections; scabies

**Table 2:** List of the investigated usnic acid-loaded liposomes.

Potential Therapy	Composition	Particle Size (nm)	EE (%)	Stability	Purpose	Ref.
Pulmonary tuberculosis	PC, Chol, SA	90 ± 20	99.6 ± 0.2	24 months	Enhancement of cellular penetration/uptake to macrophages and antimicrobial activity against Mbt	[15]
Pulmonary tuberculosis	PC, Chol, SA	146 ± 1.91	99.56 ± 0.74	n.a.	Enhancement of antimicrobial activity against clinical isolates of Mbt alone or in combination with antituberculosis drugs (RIF and INH)	[16]
Infections caused by MRSA	PC, Chol, SA, β-CD	n.a.	99.5 ± 0.2	5 months	Increase of solubility and targeted delivery for exploiting the antibacterial activity against MRSA	[17]
Infections caused by MRSA	PC, Chol, SA	121.3 ± 1.8	99.0 ± 2.7%,	n.a.	Enhancement of antimicrobial activity against MRSA alone or in combination with vancomycin	[18]
(medical device-related) infections caused by *S. epidermidis*	DOPC, Chol and one of the three GAs (GA 1, 2 and 3) or CTAB	∼100	from 12 to 89	At least two weeks	Promotion of (+)-usnic acid penetration in biofilm matrix with a consequent increase of antimicrobial activity against *S. epidermidis*	[19]
Oxidative stress-related diseases	DMPC, Chol and L-prolinol derived surfactants	∼100	from 52 ± 2 to 77 ± 2	Aggregation after two weeks	Enhancement of antioxidant properties of (+)-usnic acid	[20]
Dressing for burn wounds *	PC, collagen, PEG/PG	70	n.a.	n.a.	Burn wounds healing	[21,22]
Dressing for burn wounds **	PC, gelatin, PG	n.a.	n.a.	n.a.	Burn wounds healing	[23]

EE: entrapment efficiency; Mbt: *Mycobacterium tuberculosis*; n.a.: not applicable; MRSA: methicillin-resistant *Staphylococcus aureus*; PC: soya phosphatidylcholine; Chol: cholesterol; SA: stearylamine; β-CD: β-cyclodextrin; RIF: rifampicin; INH: isoniazid; DOPC: 1,2-dioleoyl-sn-glycero-3-phospholcholine; GA: glucosylated amphiphiles; CTAB: cetyltrymethyl ammonium bromide; DMPC:1,2-dimyristoyl-sn-glycero-3-phosphocholine. PEG: polyethylene glycol; PG: propylene glycol; * PC liposomes with usnic acid incorporated into thin film based on collagen; ** PC liposomes with usnic acid incorporated into thin film based on gelatin.

**Table 3:** List of the investigated usnic acid-loaded polymeric nano- and microcarriers.

Potential Therapy	Composition	Particle Size	EE (%)	Stability	Purpose	Ref.
**Polymeric Nanoparticles**						
**Nanocapsules**						
Sarcoma	PLGA, soybean oil, PC, poloxamer F68	324 ± 88 nm	99.4 ± 0.2	36 months	Enhancement of antitumor activity and reduction of hepatotoxicity	[31,32]
**Nanospheres**						
Lung cancer	Heparin–ADH–GG copolymer, Pluronic F 68	83.38 ± 1.18nm	99.98 ± 0.50	n.a.	Site-specific delivery in the cancer cells	[33]
**Polymeric Nanofibers**						
Diabetic wound healing treatment	Eudragit L100 or PVP	n.a.	n.a.	n.a.	Improvement of antimicrobial activity against *S. aureus*	[34]
**Polymeric Microparticles**						
Sarcoma	PLGA, PEG, PVA, Poloxamer F68	7.02 ± 2.74 μm	∼100	7 months	Enhancement of antitumor activity and reduction of hepatotoxicity	[35]
Inflammation	PCL, PEG, PVA	13.54 μm	97.72	n.a.	Enhancement of anti-inflammatory activity and reduction of acute toxicity	[36]
Infected chronic wounds	CPLLA	2.4 ± 0.8 μm	80	n.a.	Improvement of antibiofilm activity against *S. epidermidis*	[37]
Wounds and burns, implanted device-related infections *	PLA, PVA, Ti	5 µm	n.a.	n.a.	Improvement of antibiofilm activity against *S. aureus*	[38]
Wounds and burns, implanted device-related infections **	PLGA, PVA, Fe_3_O_4_, myristic acid, Ti	5–20 µm	n.a.	n.a.	Improvement of antibiofilm activity against *S. aureus*	[39]

EE: entrapment efficiency; PLGA: Poly (d,l-lactic-acid-*co*-glycolic acid) polymer; PC: soybean phosphatidylcholine; ADH: adipic acid dihydrazide; GG: gellan gum; n.a.: not applicable; PVP: polyvinylpyrrolidone; PEG: poly ethylene glycol; PVA: polyvinyl alcohol; PCL: poly-𝜀-caprolactone; CPLLA: carboxylated poly(l-lactide); Ti: Titanium;* thin film based on PLA-PVA microspheres deposited to Ti substrates.** thin film based on PLGA-PVA microspheres containing Fe_3_O_4_ nanoparticles coated with myristic acid deposited to Ti substrates.

## References

[1] Galanty A., Pasko P., Podolak I. (2019). Enantioselective activity of usnic acid: A comprehensive review and future perspectives. Phytochem. Rev..

[2] Žugić A., Isaković A., Jeremić I., Tadic V. (2019). Cytotoxic activity of supercritical CO_2_ extract of old man’s beard in L929 fibrosarcoma cell line. Lek. Sirovine.

[3] Zugic A., Jeremic I., Isakovic A., Arsic I., Savic S., Tadic V. (2016). Evaluation of anticancer and antioxidant activity of a commercially available CO_2_ supercritical extract of old man’s beard (Usnea barbata). PLoS ONE.

[4] Zugic A., Lunter D.J., Daniels R., Pantelic I., Tasic Kostov M., Tadic V., Misic D., Arsic I., Savic S. (2016). Usnea barbata CO_2_-supercritical extract in alkyl polyglucoside-based emulsion systems: Contribution of Confocal Raman imaging to the formulation development of a natural product. Pharm. Dev. Technol..

[5] Žugić A.R., Lukić M.Z., Tasić Kostov M.Z., Tadić V.M., Arsić I.A., Mišić D.R., Petrović S.D., Savić S.D. (2015). Alkyl polyglucoside-stabilized emulsion as a prospective vehicle for Usnea barbata CO_2_ supercritical extract: Assessing stability, safety and efficiency of a topical formulation. Hem. Ind..

[6] Sokolov D.N., Luzina O.A., Salakhutdinov N.F. (2012). Usnic acid: Preparation, structure, properties and chemical transformations. Russ. Chem. Rev..

[7] Araújo A.A.S., de Melo M.G.D., Rabelo T.K., Nunes P.S., Santos S.L., Serafini M.R., Santos M.R.V., Quintans-Júnior L.J., Gelainc D.P. (2015). Review of the biological properties and toxicity of usnic acid. Nat. Prod. Res..

[8] Luzina O.A., Salakhutdinov N.F. (2018). Usnic acid and its derivatives for pharmaceutical use: A patent review (2000-2017). Expert Opin. Pat..

[9] Nikolic I., Mitsou E., Pantelic I., Randjelovic D., Markovic B., Papadimitriou V., Xenakis A., Lunter D.J., Zugic A., Savic S. (2020). Microstructure and biopharmaceutical performances of curcumin-loaded low-energy nanoemulsions containing eucalyptol and pinene: Terpenes’ role overcome penetration enhancement effect?. Eur. J. Pharm. Sci..

[10] Nikolic I., Lunter D.J., Randjelovic D., Zugic A., Tadic V., Markovic B., Cekic N., Zivkovic L., Topalovic D., Spremo-Potparevic B. (2018). Curcumin-loaded low-energy nanoemulsions as a prototype of multifunctional vehicles for different administration routes: Physicochemical and in vitro peculiarities important for dermal application. Int. J. Pharm..

[11] (2009). Martindale: The Extra Pharmacopoeia.

[12] Drugs. http://www.drugs.com.

[13] Natarajan J.V., Nugraha C., Ng X.W., Venkatraman S. (2014). Sustained-release from nanocarriers: A review. J. Control Release.

[14] Sawant R.R., Torchilin V.P. (2010). Liposomes as ‘smart’ pharmaceutical nanocarriers. Soft Matter.

[15] Lira M.C.B., Siqueira-Moura M.P., Rolim-Santos H.M.L., Galetti F.C.S., Simioni A.R., Santos N.P., Egito E.S.T., Silva C.L., Tedesco A.C., Santos-Magalhaes N.S. (2009). In vitro uptake and antimycobacterial activity of liposomal usnic acid formulation. J. Liposome Res..

[16] Ferraz-Carvalho R.S., Pereira M.A., Linhares L.A., Lira-Nogueira1 M.B.C., Cavalcanti I.M.F., Santos-Magalhães N.S., Montenegro L.M.L. (2016). Effects of the encapsulation of usnic acid into liposomes and interactions with antituberculous agents against multidrug-resistant tuberculosis clinical isolates. Mem. Inst. Oswaldo Cruz.

[17] Lira M.B.C., Ferraz M.S., da Silva D.G.V.C., Cortes M.E., Teixeira K.I., Caetano N.P., Sinisterra R.B., Ponchel G., Santos-Magalhaes N.S. (2009). Inclusion complex of usnic acid with β-cyclodextrin: Characterization and nanoencapsulation into liposomes. J. Incl. Phenom. Macrocycl. Chem..

[18] Cavalcanti I.M.F., Menezes T.G.C., de Almeida Campos L.A., Ferraz M.S., Maciel M.A.V., Caetano M.N.P., Santos-Magalhaes N.S. (2018). Interaction study between vancomycin and liposomes containing natural compounds against methicillin-resistant Staphylococcus aureus clinical isolates. Braz. J. Pharm. Sci..

[19] Francolini I., Giansanti L., Piozzi A., Altieri B., Mauceri A., Mancini G. (2019). Glucosylated liposomes as drug delivery systems of usnic acid to address bacterial infections. Colloid Surf. B.

[20] Battista S., Campitelli P., Galantini L., Köber M., Vargas-Nadal G., Ventosa N., Giansanti L. (2020). Use of N-oxide and cationic surfactants to enhance antioxidant properties of (+)-usnic acid loaded liposomes. Colloid Surf. A.

[21] Nunes P.S., Bezerra M.S., Costa L.P., Cardoso J.C., Albuquerque R.L.C., Rodrigues M.O., Barin G.B., da Silva F.A., Araujo A.A.S. (2010). Thermal characterization of usnic acid/collagen-based films. J. Anal. Calorim..

[22] Nunes P.S., Albuquerque R.L.C., Cavalcante D.R.R., Dantas M.D.M., Cardoso J.C., Bezerra M.S., Souza J.C.C., Serafini M.R., Quitans L.J., Bonjardim L.R. (2011). Collagen-based films containing liposome-loaded usnic acid as dressing for dermal burn healing. Biomed. Res. Int..

[23] Nunes P.S., Rabelo A.S., Souza J.C., Santana B.V., da Silva T.M., Serafini M.R., Dos Passos Menezes P., Dos Santos Lima B., Cardoso J.C., Alves J.C. (2016). Gelatin-based membrane containing usnic acid-loaded liposome improves dermal burn healing in a porcine model. Int. J. Pharm..

[24] Mukerjee A., Pandey H., Tripathi A.K., Singh S.K. (2019). Development, characterization and evaluation of cinnamon oil and usnic acid blended nanoemulsion to attenuate skin carcinogenicity in swiss albino mice. Biocatal. Agric. Biotechnol..

[25] Nascimento Porto Neto A., Santos Cruz C.F., Serafini M.R., Passos Menezes P., Carvalho Y.M.B.G., Santos Matos C.R., Santos Nunes P., Cardoso J.C., Albuquerque Júnior R.L.C., Rolim Neto P.J. (2017). Usnic acid-incorporated alginate and gelatin sponges prepared by freeze-drying for biomedical applications. J. Anal. Calorim..

[26] Santos M.R., Alcaraz-Espinoza J.J., Costa M.M., Oliveira H.P. (2018). Usnic acid-loaded polyaniline/polyurethane foam wound dressing: Preparation and bactericidal activity. Mater. Sci. Eng. C.

[27] Ficai D., Ardelean J.L., Holban A.M., Diţu L.M., Gudovan D., Sönmez M., Truşcă R., Kaya A., Ficai A., Andronescu E. (2018). Manufacturing nanostructured chitosan-based 2D sheets with prolonged antimicrobial activity. Rom. J. Morphol. Embryol..

[28] Dasgupta Q., Madras G., Chatterjee K. (2017). Controlled release of usnic acid from biodegradable polyesters to inhibit biofilm formation. ACS Biomater. Sci. Eng..

[29] Karabacak R.B., Tay T., Kıvanc M. (2014). Preparation of novel antimicrobial polymer colloids based on (+)-usnic acid and poly(vinylbenzyl chloride). React. Funct. Polym..

[30] Mishra B., Patel B.B., Tiwari S. (2010). Colloidal nanocarriers: A review on formulation technology, types and applications toward targeted drug delivery. Nanomed. Nanotechnol..

[31] Santos N.P., Nascimento S.C., Silva J.F., Pereira E.C.G., Silva N.H., Honda N.K., Santos-Magalhaes N.S. (2005). Usnic acid-loaded nanocapsules: An evaluation of cytotoxicity. J. Drug Deliv. Sci. Technol..

[32] Da Silva Santos N.P., Nascimento S.C., Wanderley M.S.O., Pontes N.T., da Silva J.F., de Castro C.M.M.B., Pereira E.C., da Silva N.H., Honda N.K., Santos-Magalhaes N.S. (2006). Nanoencapsulation of usnic acid: An attempt to improve antitumour activity and reduce hepatotoxicity. Eur. J. Pharm. Biopharm..

[33] Garg A., Garg S., Sahu N.K., Rani S., Gupta U., Yadav A.K. (2019). Heparin appended ADH-anionic polysaccharide nanoparticles for site-specific delivery of usnic acid. Int. J. Pharm..

[34] Araujo E.S., Eugênia C.P., da Costa M.M., da Silva N.H., de Oliveira H.P. (2016). Bactericidal activity of usnic acid-loaded electrospun fibers. Recent Pat. Nanotechnol..

[35] Ribeiro-Costa R.M., Alves A.J., Santos N.P., Nascimento S.C., Goncalves E.C.P., Silva N.H., Honda N.K., Santos-Magalhaes N.S. (2004). In vitro and in vivo properties of usnic acid encapsulated into PLGA-microspheres. J. Microencapsul..

[36] Barbosa J.A.P., Franco E.S., Silva C.V.N.S., Bezerra T.O., Santana M.A.N., Junior C.H.R.C., Silva T.G., Santos N.P.S., Maia M.B.S. (2017). Poly-𝜀-caprolactone microsphere polymers containing usnic acid: Acute toxicity and anti-inflammatory activity. Evid.-Based Complementary Altern..

[37] Martinelli A., Bakry A., D’Ilario L., Francolini I., Piozzi A., Taresco V. (2014). Release behavior and antibiofilm activity of usnic acid-loaded carboxylated poly(L-lactide) microparticles. Eur. J. Pharm. Biopharm..

[38] Grumezescu V., Socol G., Grumezescu A.M., Holban A.M., Ficai A., Trusca R., Bleotu C., Balaure P.C., Cristescu R., Chifiriuc M.C. (2014). Functionalized antibiofilm thin coatings based on PLA–PVA microspheres loaded with usnic acid natural compounds fabricated by MAPLE. Appl. Surf. Sci..

[39] Grumezescu V., Holban A.M., Grumezescu A.M., Socol G., Ficai A., Vasile B.S., Trusca R., Bleotu C., Lazar V., Chifiriuc C.M. (2014). Usnic acid-loaded biocompatible magnetic PLGA-PVA microsphere thin films fabricated by MAPLE with increased resistance to staphylococcal colonization. Biofabrication.

[40] Marinho K.S.N., Antonio E.A., Silva C.V.N.S., Silva K.T.D., Teixeira V.W., de Aguiar Junior F.C.A., Santos K.R.P.D., Silva N.H.D., Santos N.P.S. (2017). Hepatic toxicity caused by PLGA-microspheres containing usnic acid from the lichen Cladonia substellata (AHTI) during pregnancy in Wistar rats. Acad. Bras. Cienc..

[41] Taresco V., Francolini I., Padella F., Bellusci M., Boni A., Innocenti C., Martinelli A., D’Ilario L., Piozzi A. (2015). Design and characterization of antimicrobial usnic acid loaded-core/shell magnetic nanoparticles. Mater. Sci. Eng. C.

[42] Grumezescu A.M., Cotar A.I., Andronescu E., Ficai A., Ghitulica C.D., Grumezescu V., Vasile B.S., Chifiriuc M.C. (2013). In vitro activity of the new water-dispersible Fe_3_O_4_@ usnic acid nanostructure against planktonic and sessile bacterial cells. J. Nanopart. Res..

[43] Grumezescu A.M., Saviuc C., Chifiriuc M.C., Hristu R., Mihaiescu D.E., Balaure P., Stanciu G., Lazar V. (2011). Inhibitory activity of Fe_3_O_4_/oleic acid/usnic acid-core/shell/extrashell nanofluid on *S. aureus* biofilm development. IEEE Trans. Nanobiosci..

[44] Grumezescu A.M., Holban A.M., Andronescu E., Mogosanu G.D., Vasile B.S., Chifiriuc M.C., Lazar V., Andrei E., Constantinescu A., Maniu H. (2014). Anionic polymers and 10 nm Fe_3_O_4_@UA wound dressings support human foetal stem cells normal development and exhibit great antimicrobial properties. Int. J. Pharm..

[45] Holban A.M., Grumezescu A.M., Andronescu E., Grumezescu V., Chifiriuc C.M., Radulescu R. (2013). Magnetite-usnic acid nanostructured bioactive material with antimicrobial activity. Rev. Rom. Mater..

[46] Alpsoy L., Baykal A., Amir M.d., Ulker Z., Nawaz M. (2018). SPION@APTES@FA-PEG@usnic acid bionanodrug for cancer therapy. J. Supercond. Nov. Magn. May.

[47] Akal Z.U., Alpsoy L., Baykal A. (2016). Superparamagnetic iron oxide conjugated with folic acid and carboxylate quercetin for chemotherapy applications. Ceram. Int..

[48] Garg S., Garg A., Sahu N.K., Yadav A.K. (2019). Synthesis and characterization of nanodiamond-anticancer drug conjugates for tumor targeting. Diam. Relat. Mater..

